# pH-Sensitive Chitosan-Based Hydrogels Trap Poloxamer Micelles as a Dual-Encapsulating Responsive System for the Loading and Delivery of Curcumin

**DOI:** 10.3390/polym17101335

**Published:** 2025-05-14

**Authors:** Alejandra E. Herrera-Alonso, Daniela F. Rodríguez-Chávez, Alberto Toxqui-Terán, José F. Rubio-Valle, José E. Martín-Alfonso, Samuel Longoria-García, Hugo L. Gallardo-Blanco, Celia N. Sánchez-Domínguez, Margarita Sánchez-Domínguez

**Affiliations:** 1 Centro de Investigación en Materiales Avanzados, S.C. (CIMAV), Unidad Monterrey, Alianza Norte 202, Parque de Investigación e Innovación Tecnológica, Ciudad Apodaca 66628, Mexico; alejandra.herrera@cimav.edu.mx (A.E.H.-A.); daniela.rodriguez@cimav.edu.mx (D.F.R.-C.); atoxquiteran@gmail.com (A.T.-T.); 2 Pro2TecS—Chemical Process and Product Technology Research Center, Department of Chemical Engineering and Materials Science, Universidad de Huelva, ETSI, Campus de “El Carmen”, 21071 Huelva, Spain; josefernando.rubio@diq.uhu.es; 3 Departamento de Bioquímica y Medicina Molecular, Facultad de Medicina, Universidad Autónoma de Nuevo León, Monterrey 64460, Mexico; samuel.longoriagr@uanl.edu.mx (S.L.-G.); celia.sanchezdm@uanl.edu.mx (C.N.S.-D.); 4 Servicio de Oncología, Centro Universitario Contra el Cáncer (CUCC), Hospital Universitario “Dr. José Eleuterio González”, Universidad Autónoma de Nuevo León, Monterrey 64451, Mexico; hugo.gallardobl@uanl.edu.mx

**Keywords:** hydrogel, poloxamer micelle, curcumin, pH-responsive hydrogel, rheology

## Abstract

pH-sensitive hydrogels are important soft biomaterials as they mimic biological organisms by altering their properties in response to small pH changes in biological fluids. In this work, novel chitosan (Cs) hydrogels were developed using an innovative dual curcumin (Cur) encapsulation system. Cur was loaded into poloxamer 407 micelles and incorporated into citric acid (CA) cross-linked Cs hydrogels using a central composite design. The hydrogels were characterized using Fourier transform infrared spectroscopy (FTIR), scanning electron microscopy (SEM), differential scanning calorimetry (DSC), rheological tests, and in vitro experiments, such as hemolysis and cytotoxicity assays. FTIR confirmed cross-linking between Cs and CA, while DSC suggested interactions between Cur-loaded micelles and the hydrogel matrix. Rheological analysis revealed gel-like behavior, with G′ consistently higher than G, and temperature influenced hydrogel properties. SEM showed a denser network when Cur-loaded micelles were incorporated, slowing Cur release. At physiological pH (7.4), 75% of Cur was released after 7 days, while 84% was released at pH 5.5, showing pH-responsive behavior. Cytotoxicity tests showed over 80% viability of VERO CCL-81 cells (0.2–20 ppm hydrogel). This dual-encapsulation system provides a simple and effective platform for loading lipophilic drugs into pH-responsive hydrogels.

## 1. Introduction

Hydrogels are hydrophilic polymeric networks capable of absorbing, retaining, and releasing substantial amounts of water or water-soluble molecules, including biological compounds, drugs, and proteins [1,2]. The incorporation of particles loaded with drugs into hydrogels can provide the matrix with a second barrier that reduces diffusion [3]. Hydrogels are considered potential drug nanocarriers for use as targeted release systems, tissue engineering, and wound healing [4]. Among these, pH-sensitive hydrogels occupy a special space because they mimic the behavior of living organisms by changing their inherent properties in response to small changes in the pH of biological fluids [5]. Several materials are being explored for hydrogel preparation, and chitosan (Cs) is a prominent candidate [6]. This is a cationic biopolymer composed of N-acetyl-D-glucosamine and D-glucosamine units linked by β-(1→4)-glycosidic bonds [7]. Cs forms gels easily at concentrations above 1 wt% [8]; however, cross-linking with various compounds like genipin, 1,4-butanediol diglycidyl ether, and glyoxal [1] allows for gel formation even at lower concentration. The use of cross-linking agents such as epichlorohydrin, diisocyanate, and glutaraldehyde has decreased because of their cytotoxicity [9]. Compounds of natural origin like citric acid (CA) are of interest for polysaccharide modification and cross-linking because they are non-toxic [10,11].

Cs exhibits biocompatibility, biodegradability, mucoadhesiveness, and antimicrobial activity, along with low immunogenicity, and it can enhance the absorption of drugs across biological membranes. All these properties make Cs appropriate for biomedical applications [10,12], for example, as a vehicle for oral drug delivery and wound healing [13], targeting infected sites while minimizing systemic side effects. If appropriately formulated, Cs hydrogels may also have the potential to improve the delivery and bioavailability [11] of active pharmaceutical ingredients which are difficult to incorporate into aqueous systems, such as curcumin (Cur) (diferuloylmethane, 1,7-bis(4-hydroxy-3-methoxyphenyl)-1-6-heptadiene-3,5-dione). Cur is an active ingredient extracted from the *Curcuma longa* plant, and it has been studied for its anti-inflammatory, antioxidant, antibacterial, and anticancer properties [14,15,16]. In topical administration, it could stimulate wound healing, protecting the skin from infections by reducing bacterial resistance [17]. A challenge of the encapsulation and delivery of Cur is its low solubility in water (0.6 µg/mL) [18], and although its solubility increases at alkaline pH, it is susceptible to degradation under such conditions [19]. Hence, the bioavailability of this compound at physiological pH is very low, and this makes Cur difficult to use in medical applications [20,21,22]. One possible option is to encapsulate it within a system with a hydrophobic core and a hydrophilic shell, such as polymer micelles [23,24]. Poloxamers are amphiphilic block copolymers of poly (ethylene oxide) (PEO) and poly (propylene oxide) (PPO) (PEO-PPO-PEO) [25]; the advantages of using such polymeric micelles include their stability in the bloodstream, their ability to penetrate through vascular tissues towards tumor sites, and their ability to accumulate by size or by incorporation of specific ligands, allowing for enhanced permeability and retention (EPR) effects [26].

Incorporating Cur directly into a Cs hydrogel results in poor dispersion and reduced bioavailability due to curcumin’s hydrophobicity. However, pre-encapsulating curcumin within poloxamer micelles prior to hydrogel integration enables the formation of a homogeneous dual-encapsulation system, improving solubility and controlled release properties [27]. Previous studies have explored similar strategies for sustained drug release. For instance, Alibolandi et al. [28] encapsulated Cur within poly(ethylene glycol)-block-polylactic acid (PEG-PLA) micelles and incorporated them into dextran hydrogels, achieving a release of ~60% over five days due to the high viscosity of the hydrogel matrix. Similarly, Zhao et al. [29] developed a thermosensitive hydrogel composed of poloxamer 407, Cs, and hyaluronic acid loaded with dihydromyricetin, demonstrating drug release over 100 h in PBS at 37 °C, reducing the frequency of required treatments.

Given the therapeutic potential of Cur and the challenges associated with its solubilization, stability, and sustained release, to overcome these challenges, this work aims to develop a novel dual-encapsulation system. By loading curcumin into poloxamer 407 micelles and incorporating these into Cs hydrogels cross-linked with a biocompatible agent such as citric acid, this dual-encapsulation strategy ensures pH-responsive and sustained drug release under physiological conditions (pH 7.4), with accelerated release in mildly acidic environments (pH 5.5). This water-based drug delivery system offers a highly bioavailable and controlled-release platform for lipophilic active ingredients, surpassing conventional hydrogel-based formulations limitations that rely on the suspension of insoluble drug particles, thereby offering a more effective and controlled therapeutic strategy.

## 2. Experimental

### 2.1. Materials

Low-molecular-weight chitosan (50,000–190,000 g/mol), with an 84% degree of deacetylation and Poly (ethylene glycol)-block-poly (propylene glycol)-block-poly (ethylene glycol) (Poloxamer 407 (P407), Pluronic^®^ F-127), was purchased from Sigma Aldrich (Saint Louis, MO, USA) and used with any other treatment; these materials were employed as raw materials to produce hydrogels loaded with micelles encapsulating curcumin (Cur). Citric acid (CA) (>97% purity) was used as a cross-linking agent. Cur (>65% purity) from turmeric powder was used as a model lipophilic drug. D-α-tocopherol polyethylene glycol succinate 1000 (TPGS, 99.5% purity) was used to enhance drug solubility for release quantification. Acetone (99.5% purity) and glacial acetic acid (99% purity) were employed without further treatment. Phosphate-buffered saline (PBS) powder (pH 7.4) and Dulbecco’s Modified Eagle Medium (DMEM), with 4.5 g/L od D-Glucose and 110 mg/L of sodium pyruvate, supplemented with amphotericin B as an antifungal and streptomycin/penicillin as an antibiotic (0.1%), as well as (3-(4,5-dimethylthiazol-2-yl)-2,5-diphenyltetrazolium bromide (MTT reagent), were used for the cytotoxicity tests. All reagents were purchased from Sigma-Aldrich (Saint Louis, MO, USA), unless otherwise stated.

### 2.2. Formulation of Cross-Linked Chitosan Hydrogels Charged with Cur-Loaded P407 Micelles

Cur-loaded P407 micelle preparation. P-407 micelles loaded with Cur were formulated using the film hydration method [30]. Briefly, the surfactant and the active pharmaceutical ingredient were solubilized in acetone, and then the solvent was removed by rotary evaporation using IKA RV10 equipment (IKA-Werke GmbH & Co. KG, Staufen im Breisgau, BW, Germany). The remaining film was rehydrated to achieve a P-407 concentration of 12.5 wt%, dissolving the Cur-loaded P407 in deionized water, followed by freeze-drying in Labconco equipment (Thermo Fisher Scientific, Kansas City, MI, USA). For the cross-linking of Cs, the reaction mixtures were prepared in 0.05 M CH_3_COOH with different concentrations of Cs (0.5, 0.75 and 1 wt%) and CA (0.05, 0.075 and 0.1 wt%), and at a constant concentration of Cur-loaded P407 micelles (1.18 wt%, to obtain a concentration of 0.013 wt% of Cur in the reaction mixtures). The mixtures were stirred for 6–24 h. The resulting samples were freeze-dried. Finally, the freeze-dried Cur composites were rehydrated at a 1:1 weight ratio of freeze-dried sample to water, yielding hydrogels with approximately 100% swelling. The reaction conditions for cross-linking are presented in Table 1.

### 2.3. Curcumin Encapsulation Efficiency (%Cur EE) and Loading Capacity (%Cur LC)

**Figure A1:**
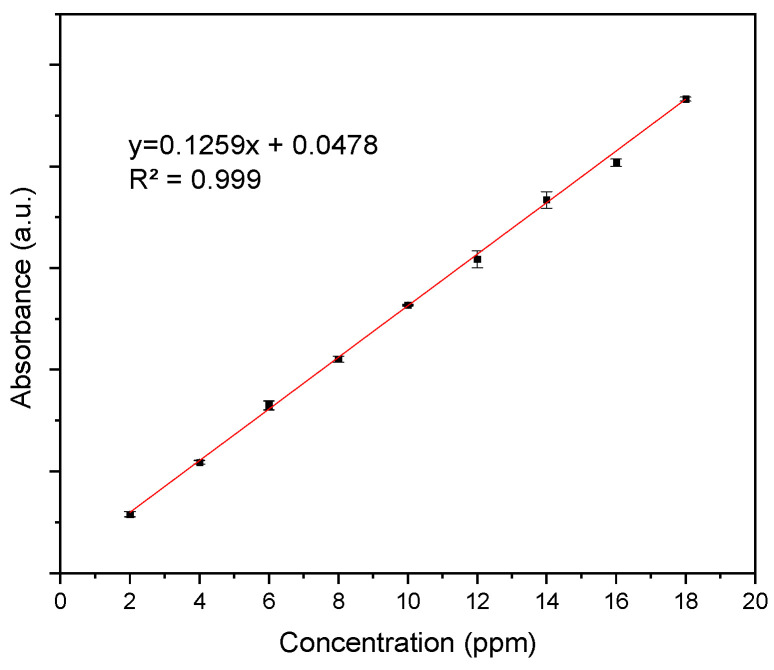
Calibration curve of curcumin.

The percentage of encapsulation efficiency (*%Cur EE*) allowed for the calculation of the difference between the theoretical amount of Cur present in the hydrogels and the actual amount. Encapsulation efficiency was determined by taking a certain amount of freeze-dried gel containing a theoretical maximum of the active ingredient that was weighed and dissolved in a 0.05 M CH_3_COOH solution; the absorbance of the samples was measured using a Mecasys Optizen Pop Uv-Vis spectrophotometer (Daejeon, Seoul, South Korea) at 435 nm. The concentration of Cur used to assess the percentage of encapsulation efficiency was calculated using a linear equation, which was previously obtained from a calibration curve of absorbance of Cur at 435 nm vs. Cur concentration, which is shown in Appendix A in Figure A1. The following Equation (1) was used to determine the %*Cur EE* (percentage of Cur encapsulation efficiency), where *W_i_* is the actual amount of active ingredient and *W_t_* is the theoretical amount of Cur in the sample. The incorporation of Cur into the hydrogels was evaluated in triplicate [8].

(1) $$\%CurEE=\left[W_{i}\times 100\right]/W_{t}$$

Also, the maximum loading capacity of Cur in the hydrogels was determined using Equation (2)

(2) $$\%CurLC=\left[W_{rlc}\times 100\right]/W_{th}$$

where *W_rlc_* is the real weight of Cur loaded into the hydrogels, and *W_th_* is the total weight of components in the hydrogels (P407, Cs, CA, Cur, and water).

The percentage of curcumin loading capacity in poloxamer micelles (%*Cur LC_m_*) was calculated using the equation

(3) $$\%Cur\;{LC}_{m}=\left[W_{lcm}\times \%{EE}_{m}\right]\;/\;W_{tm}$$

where *LC_m_* is the loading capacity of curcumin in the micelles, *W_lcm_* is the weight of curcumin added into the micelles, %*EE_m_* is the percentage of Cur encapsulation efficiency in the micelles, and *W_tm_* is the total weight of the micelle system (P407 + Cur). Meanwhile, for the calculation of the percentage of the encapsulation efficiency of curcumin in the micelles (%*Cur EE_m_*), we used the equation

(4) $$\%Cur\;{EE}_{m}=\left[W_{im}\times 100\right]/\;W_{tm}$$

where *W_im_* is the real amount of active ingredient in the micelles and *W_tm_* is the theoretical total amount of curcumin in the micelles. 

### 2.4. Characterization Techniques

#### 2.4.1. Fourier Transform Infrared Spectroscopy (FTIR-ATR)

The chemical structure of the polymers and hydrogels was analyzed using a Nicolet iS50 FT-IR spectrometer, which was equipped with a universal ATR monolithic diamond crystal sampling accessory (Thermo Fisher Scientific, Madison, WI, USA). The attenuated total reflection Fourier transform infrared spectroscopy (ATR-FTIR) was recorded in the 4000 to 400 cm^−1^ wavenumber range, with an average of 32 scans taken at a resolution of 4 cm^−1^ ± 0.01 cm^−1^ per sample cycle at room temperature, and the background spectra were automatically subtracted. Reference spectra were obtained using a blank ATR crystal after cleaning the ATR module and prior to applying the sample. The ATR element and pressure clamp were cleaned several times with methanol and dried. A small amount of the solid sample or a drop of the liquid sample was placed directly on the flat surface of the monolithic diamond crystal cell. The solid sample was pressed using a clamp, with manual adjustment of the applied compression force applied, while the liquid sample was neither covered nor pressed. All measurements were carried out using the same instrument settings.

#### 2.4.2. Differential Scanning Calorimetry (DSC)

Conventional DSC analysis and Modulated Differential Scanning Calorimetry (MDSC) were performed with the TA Instrument DSC Q200 (Lindon, UT, USA), using 8–10 mg of the hydrogel blank (Cs cross-linked with CA (Cs-CA) and hydrogel charged with Cur-loaded P407 micelles (AC3). The samples were pressed into aluminum pans and subjected to the same measurement procedure: a temperature interval between −60 and 65 °C; for MDSC, cooling to −60 °C was carried out with a heating rate of 10 °C/min; and nitrogen was used as a purge gas at a flow rate of 50 mL/min with 40 s modulation and 1.5 °C amplitude. The cooling temperature was left for 10 min before starting heating to 65 °C at a rate of 10 °C/min. After the DSC measurement, the pan was weighed to verify that it had been sealed correctly, and that no water had evaporated.

#### 2.4.3. Scanning Electron Microscopy (SEM)

The morphology of the hydrogels was characterized using a Jeol 6010 Plus field emission scanning electron microscope (Akishima, Tokyo, Japan). The samples were freeze-dried and cold cross-cut with a razor blade for evaluation according to Park et al. [31]. The analyses were carried out using backscattered electrons at 15 kV acceleration voltage.

#### 2.4.4. Rheological Characterization

Rheological measurements of hydrogels were carried out using an AR G2-controlled stress rheometer (TA Instruments, New Castle, DE, USA) using a smooth steel parallel plate (40 mm diameter, 1 mm gap). Small-amplitude oscillatory shear (SAOS) measurements, inside the linear viscoelasticity regime, were performed in a frequency range between 0.1 and 100 rad/s, at 25 °C. Strain sweep tests, at a frequency of 1 Hz, were previously performed to determine the linear viscoelastic regime. Viscous flow measurements were performed in a shear rate range of 0.01–100 s^−1^. Flow curves were described well by the power law model (R^2^ ˃ 0.995)

(5) $$\eta =k{\left(\dot{\gamma }\right)}^{n}$$

where η is the non-Newtonian viscosity; $\dot{\gamma }$ is the shear rate; *n* is a parameter related to the slope of the shear-thinning region; and k is a parameter related to the consistency of the hydrogels. All tests were performed in triplicate, at the preparation temperature (22 °C), as well as at the controlled release assay temperature (37 °C).

#### 2.4.5. Cur Release from the Hydrogels

A system of three vertical Franz-type diffusion cells (PermeGear, Inc., Hellertown, PA, USA) was used to evaluate Cur release from the hydrogel systems and curcumin diffusion through the hydrogel and membrane. Each experiment was run in triplicate. For each run, 20 mg of freeze-dried AC3 hydrogel was hydrated with 20 mg of deionized water. The 40 mg hydrogel sample (containing 0.116 mg of Cur) was placed on top of a PVDF membrane (0.22 μm pore size, diameter 47 mm), the system was jacketed, and the temperature was controlled at 37 °C. The acceptor medium consisted of 8 mL of PBS; release experiments were run at two different pH conditions (pH 7.4 and 5.5). Such pH conditions were chosen for this study since the intended application is topical and the pH of the skin tissue typically ranges between 4.1 and 5.8 [32], in contrast to physiological pH (7.4). To prevent saturation of the receptor solution, the entire 8 mL measure of acceptor PBS medium was replaced with 8 mL of fresh buffer solution every 24 h at regular intervals. The collected solution was analyzed by Uv-Vis spectrophotometry at 435 nm wavelength. The results were compared with a calibration curve made previously. Equation (1) was used [33]. Several release models can be applied to polymeric systems; the Korsmeyer–Peppas model, shown in Equation (6), uses the exponent *n* to describe the release mechanism. Different values of *n* indicate different release types [34].

(6) $$M_{t}/M_{\infty }=kt^{n}$$

where *M_t_* is the amount of drug released at time *t*, *M_∞_* is the total amount of drug in the system, and *k* is a release constant dependent on the interactions between the polymer and the drug [35].

#### 2.4.6. Hemolysis Test

The hemolysis test evaluates the toxic effect of a material by measuring the percentage of red blood cell lysis, with the released hemoglobin quantified via UV-Vis spectrophotometry. A human peripheral blood sample was obtained from the Biobank Laboratory (LANBIOBAN) of the Faculty of Medicine, Universidad Autonoma de Nuevo León (UANL). The sample was processed, and erythrocytes were prepared according to standardized protocols, as previously described Longoria et al. [36]. A 1:99 erythrocyte dilution in PBS was mixed with 16 µL of hydrogel triplicates (at 0.2, 1, or 2 ppm) and incubated at 37 °C for 30 min. After centrifugation at 14,000 rpm for 3 min, hemoglobin release was measured at 415 nm using a NanoDrop spectrophotometer (Thermo Fisher Scientific, Wilmington, DE, USA) PBS was used as a negative control, and distilled water was used as a positive control. The percentage of hemolysis was calculated using Equation (7).

(7) $$\%H={[A}_{s}-A_{nc}]\times 100/{[A}_{pc}-A_{nc}]$$

where *A_s_* is the sample absorbance, *A_nc_* is the negative control absorbance, and *A_pc_* is the positive control absorbance.

#### 2.4.7. Cytotoxicity Test with VERO CCL-81 Cells (MTT Assay)

The cytotoxicity test was carried out with the VERO CCL-81 cell line (African green monkey kidney) to measure the cytotoxic effect in a healthy cell line [37]. All cells were acquired from the American Type Culture Collection (ATCC, Manassas, VA, USA). These cells were incubated with Dulbecco’s Modified Eagle Medium (DMEM) supplemented with 10% fetal bovine serum, amphotericin B, and streptomycin/penicillin (0.1%), at 37 °C and 5% CO_2_ atmosphere, for 8 days to allow for proliferation. Subsequently, cell viability was measured using the Cell Proliferation Kit I. Firstly, 10,000 Vero CCL-81 cells were seeded in a 96-well plate in a volume of 100 μL and allowed to incubate for 24 h. The freeze-dried hydrogels were diluted to the desired concentrations (2, 20, and 200 ppm). The cells and samples were incubated at 37 °C, in a 5% CO_2_ atmosphere, for 24 h. After removing the culture medium, 100 µL of MTT (3-(4,5-dimethylthiazol-2-yl)-2,5-diphenyltetrazolium) medium solution was added to each well. The 96-well plates containing the cell culture and the hydrogel samples were incubated for 3 h at 37 °C in a 5% CO_2_ atmosphere. Subsequently, 100 µL of acidified isopropanol pH = 3 was added to each well to dissolve the formazan crystals. Absorbance was measured using a Thermo Scientific Multi scan FC UV-Vis spectrophotometer (Madison, WI, USA) at wavelengths of 570 and 651 nm. Cells cultured in medium without hydrogel were used as a control (100% of viability). The percentage of cell viability of the samples was calculated using Equation (8):

(8) $$\%V={[A}_{s}\times 100]/A_{c}$$

where %*V* is the percentage of viability, *A_s_* is the sample absorbance, and *A_c_* is the control absorbance.

This study was conducted in accordance with the Declaration of Helsinki, and it was approved by the Ethics Committee of the School of Medicine, Universidad Autonoma de Nuevo Leon, with the protocol code BI22-00002 (approval date 12 April 2022).

### 2.5. Statistical Analysis

For the formulation of cross-linked chitosan hydrogels charged with Cur-loaded P407 micelles presented in Section 2.2, a central design composed of 3 factors with 1 replication and a center point was carried out using Minitab 20 software. The independent variables were the percentage of Cs in the reaction mixture, the reaction time, and the percentage of CA in the reaction mixture. The concentration of Cur-loaded P-407 micelles was kept at a constant value (1.18 wt% in the reaction mixture).

For in vitro biocompatibility tests, and in order to determine if there was a significant difference in the percentages of hemolysis and cell viability with respect to the evaluated concentrations, a single factor Analysis of Variance (ANOVA) was performed using Tukey’s multiple comparison test. Differences were considered statistically significant at *p* < 0.05. Minitab 20 and Prism Graph 7.0 software were used to perform statistical analyses. The results are presented as the mean value ± SD.

## 3. Results and Discussion

The formation of hydrogels with Cs can be achieved in different manners. One of them involves modifying the pH, since Cs is insoluble at alkaline pH, leading to precipitation and, in certain cases, the formation of hydrogels [38]; this occurs due to the deprotonation of the NH_2_ groups, leading to polymer chain contraction [39]. For example, Chatterjee et al. [40] obtained a hydrogel based on fish collagen and succinyl Cs encapsulating Cur in this way. However, the stability of Cur varies according to pH. Kharat et al. [41] evaluated the stability of Cur in aqueous solutions and emulsions, confirming that at neutral and alkaline pH, Cur is degraded by an auto-oxidation process. Therefore, to avoid the use of basic solutions and the degradation of Cur, the proposed strategy is to work under mild acidic conditions suitable for chitosan solubilization (0.05 M CH_3_COOH), whilst mixing with Cur-loaded P407 micelles, followed by cross-linking with citric acid, freeze-drying, and subsequent rehydration. The freeze-drying process includes freezing the sample, followed by sublimation of the ice under vacuum conditions to achieve the formation of a three-dimensional network. Ice crystals form during the freezing process; before freeze-drying, these ice crystals compress the chitosan chains, and when the ice sublimates, sponge-like structures are formed [42]. Furthermore, the pore size distribution can be controlled by regulating the freezing temperature and the concentration of chitosan in the solution [43]; the size and shape of the pores also depend on the size and shape of the ice crystals, which in turn may also by affected by other components of the system, such as the P407 micelles present in this system.

### 3.1. Curcumin Loading Capacity and Encapsulation Efficiency

Table 2 shows the results of %Cur incorporated in the hydrated hydrogels representing the loading capacity (*%Cur LC*). It also presents the percentage of active ingredient that was actually encapsulated into the hydrogels using different reaction conditions (%Cur encapsulation efficiency, or *%Cur EE*). The ANOVA statistical analysis applied to the central composite experimental design results revealed that the Cs (wt%) added to the formulations has a statistically significant effect (*p* < 0.05) on *%Cur EE*, which indicates that the amount of chitosan incorporated into the system significantly influences the encapsulation capacity of the compound.

Cur encapsulation efficiencies between 66 and 80% were obtained; the highest value was obtained with the hydrogels synthesized using the highest initial Cs concentration (1%) in the cross-linking reaction mixture. The highest percentage of loading capacity (0.38%) was obtained with the hydrogels, which were synthesized using the lowest initial concentration of Cs (0.5%); this is because there is less Cs in the samples, whilst the volume and concentration of Cur-loaded P407 micelles remained constant.

The trend obtained in *%Cur EE* agrees with the studies carried out by Jose et al. [9], who reported the synthesis of Cs microspheres for the loading and release of insulin. In their work, by increasing the concentration of Cs, the percentage of encapsulation efficiency was improved; this is because the higher the Cs concentration, the higher the viscosity of the solutions, which keeps the drug in the system. They observed the same effect when the percentage of cross-linking agent was increased.

Encapsulation efficiencies above 80% are expected to yield a homogeneous dosage, thereby improving the water solubility of the active principle, and making these systems good candidates for biomedical applications [16]. For the following characterizations, the samples with the highest encapsulation efficiency values and with the best gel consistency (AC2, AC3, and AC16) were chosen.

Comparison of the values of *%Cur LC* and *%Cur EE* obtained in this investigation versus results reported by other authors are presented in Table 3. Interestingly, the results obtained in this work are superior to most of the previously reported work on related hydrogels. Only the work by Alibolandi et al. [28] reached higher values of loading capacity (0.72% vs. 0.38%). However, these authors use PEG-PLA micelles for the encapsulation of curcumin; this copolymer is less common and considerably more expensive than the poloxamers used in the present work.

### 3.2. Chemical Characterization by FTIR-ATR

**Figure 1:**
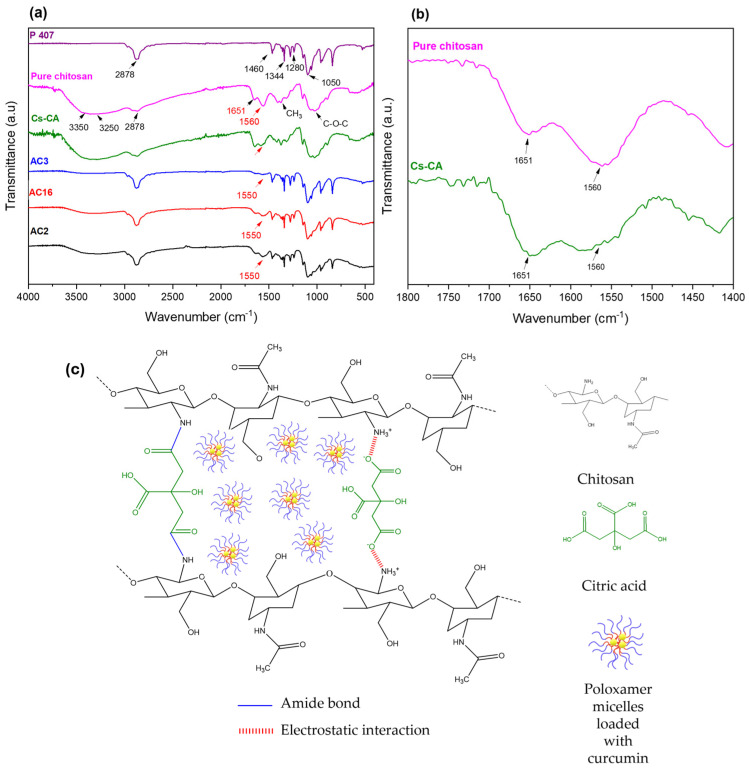
(**a**) Infrared spectra of cross-linked chitosan (Cs) with Cur-loaded P407 micelles (AC2, AC16, AC3); red arrows indicate the most significant shift corresponding to the N-H bond from the amine group due to cross-linking with CA. (**b**) Magnified view of the 1800–1400 cm^−1^ region for pure chitosan and Cs-CA spectra. (**c**) Proposed schematic representation of the cross-linked hydrogel, highlighting electrostatic interactions and amide bond formation, with Cur-loaded P407 micelles embedded in the network.

Figure 1a shows the infrared spectra of freeze-dried hydrogels AC2, AC16, and AC3, as well as the Cs-CA control blank (Cs cross-linked with CA, freeze-dried) and the raw materials P407 and Cs. A broad band is present at 3350 cm^−1^ corresponding to the O-H bond from Cs [47,48]; the band at 3250 cm^−1^ is characteristic of the N-H bond vibration of the amine [12]. The band at 2878 cm^−1^ is characteristic of C-H bond stretching; this band is present in both Cs and P407; however, its intensity is high in the AC2, AC3, and AC16 spectra, similar to the P407 spectrum; in general, in these hydrogels, the bands from P407 dominate the spectra. The bands that appear in the region between 1500 and 1700 cm^−1^ could indicate the type of cross-linking occurring between Cs and CA. Therefore, a magnified version of this region is presented in Figure 1b, it is observed that the intensity of the band at 1651 cm^−1^, characteristic of the C=O stretching of chitosan amide I group, remains similar but slightly more intense in the cross-linked sample (Cs-CA) with respect to Cs; this could be indicative of the formation of amide bonds between COOH and NH_2_ [49], whereas, compared to the hydrogel samples with Cur-loaded P407 micelles, this band decreases [50]. The band at 1570 cm^−1^, which is characteristic of the N-H stretching from the Cs amide II group, decreased in intensity from Cs to Cs-CA, which could be indicative of weak electrostatic interactions between the COO^-^ of CA and the protonated amino group of Cs in Cs-CA [49]; these changes suggest the modification of the protonated amine group of Cs by ionic cross-linking with CA [51]. With respect to hydrogels with Cur-loaded P407 micelles, this band was shifted to a lower frequency (1550 cm^−1^), and a reduction in the intensity of this band is also observed, which suggests interactions between the poloxamer molecules and cross-linked Cs hydrogel (probably hydrogen bonds) [49]. The band at around 1344 cm^−1^ is characteristic of CH_3_ group bending, and the band at 1280 cm^−1^ corresponds to the C-O bond of P407. The band at 1050 cm^−1^ is characteristic of the C-OH bond of both Cs and P407 [52]. As mentioned by Khouri et al. [53], who reported the cross-linking of chitosan films with various agents, the cross-linking of Cs with CA may not be homogeneous. Therefore, in Figure 1c, a scheme with the possible interactions between Cs and CA in the cross-linked network is proposed. Citric acid and chitosan may present an amide bond between the C of the C=O of CA and the N of the amine group of Cs [51], and/or electrostatic interactions between the COO^-^ group and the protonated amine of chitosan (NH_3_^+^) [54]. Thus, Cs can be cross-linked with citric acid either by a covalent bond via amidation or by ionic gelation due to electrostatic interactions, or both [49,53]. The physical appearance and corresponding description of selected Cs hydrogels and comparative control samples are given in Appendix A.

### 3.3. Morphology of Hydrogels

**Figure 2:**
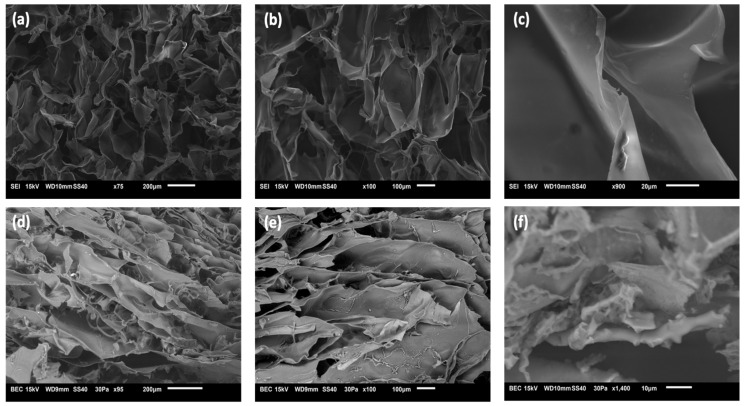
SEM micrographs of the (**a**–**c**) control blank Cs-CA and (**d**–**f**) AC3 hydrogel (75×, 95×, 100×, 900×, and 1400×).

The Cs-CA and AC3 hydrogels formed by freeze-drying and rehydration were freeze-dried again in order to be analyzed by SEM. Figure 2 shows SEM images of the control blank Cs-CA and hydrogel AC3; the images reveal hydrogel networks with pores of irregular sizes, showing that the strategy of freeze-drying a fluid dispersion of hydrogel/micelles particles, followed by rehydration (50% water), resulted in the successful formation of a continuous and dense hydrogel network. Hence, intermolecular interactions between the components were greatly enhanced by freeze-drying and rehydrating with the appropriate amount of water. Internal pores are built by swelling the polymer via the penetration of water into the interfaces of the polymer chains of both P407 and Cs during freeze-drying. As shown in Figure 2, the Cs-CA sample (without poloxamer micelles) exhibits much more open, round, and larger cells compared to the AC3 hydrogel (with curcumin-loaded poloxamer micelles), which displays a more elongated, layered structure with smaller domains. Furthermore, even the wall texture is different in both samples. In Figure 2c, it can be seen that the blank sample without poloxamer micelles, the walls are smooth and continuous with large rounded chambers, whereas Figure 2f shows that in the AC3 sample with curcumin-loaded poloxamer micelles, the texture is not smooth, as smaller pores are observed; also, in general, smaller and more elongated domains are observed. Such texture may be ascribed to the entrapment of micelles within the hydrogel. The structure and typical domain size of hydrogels play a major role in release kinetics; in general, a denser hydrogel network with smaller domains will result in a more controlled and prolonged release [55,56]. Thus, the AC3 hydrogel with curcumin incorporated in poloxamer micelles may be expected to favor a more prolonged release due to its compact structure, in addition to offering a better bioavailability of the lipophilic drug. Park et al. [31] mentioned that the hydrophobic interaction between the hydrophobic segments of poly(propylene oxide) (PPO) in the poloxamer and the chitosan cross-linking reaction allow the formation of a three-dimensional network structure. Shah et al. [17] indicated that porosity in the networks of a hydrogel facilitates the direct permeation of water, which is a desirable quality for dermatological applications, since it provides a humid environment for the skin. In our case, the texture of freeze-dried hydrogels changes considerably upon the incorporation of poloxamer micelles.

### 3.4. Differential Scanning Calorimetry (DSC)

**Figure 3:**
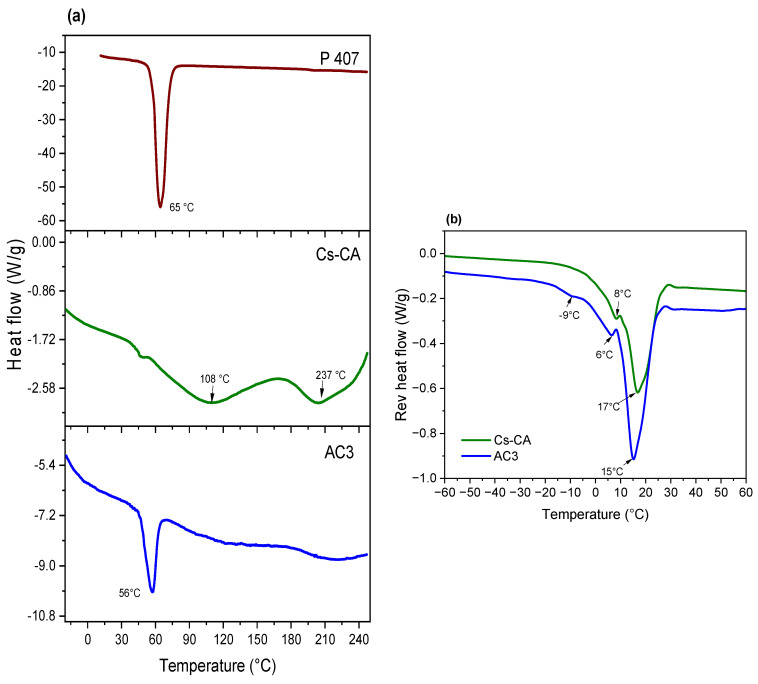
(**a**) DSC thermograms of P407, Cs-CA (rehydrated, 50% water), and AC3 (rehydrated, 50% water); (**b**) thermograms of temperature-modulated DSC (reversible heat flow) for Cs-CA and AC3 hydrogels (rehydrated, 50% water).

AC3 gel was selected to continue with the physicochemical characterizations, due to the superior physical consistency of the rehydrated gel and a higher *%Cur EE*. Figure 3 presents the DSC thermograms; for the pure P407, the melting peak is present at 65 °C; for the control hydrogel Cs-CA (rehydrated), this transition occurs at 108 °C. This indicates a slight shift with respect to the melting point of chitosan, which, according to the literature, presents a broad endothermic peak around 105 °C [57], whereas for the AC3 hydrogel (rehydrated), it occurs at 56 °C, which is closer to the melting point of P407. This difference in the melting temperature of the AC3 hydrogel with respect to P407 and Cs-CA suggests an intermolecular interaction between the cross-linked gel and the P407 micelles that alters the thermal fusion events as the composition becomes more complex [58]. The endothermic peak at 205 °C in the Cs-CA sample could be attributed to the decomposition of CA [59]; however, this peak disappears in the AC3 hydrogel sample. The same happens with the peak at 108 °C which is present in Cs-CA and absent in AC3. Once again, these changes suggest strong intermolecular interactions between the cross-linked gel network and P407 micelles in AC3, probably by hydrogen bonding [49].

Conventional DSC can provide useful information about phase transitions; however, with modulated DSC (MDSC), it is possible to investigate and differentiate thermal phenomena in which reversible and non-reversible phase transformations occur. Reversing and non-reversing signals in MDSC reveal thermodynamic and kinetic characteristics of transition, respectively [60]. In Figure 3b, reversible heat flow reveals an endotherm centered at 17 °C for Cs-CA, and at 15 °C for AC3; this may be attributed to disruption of hydrogen bonds between water and chitosan (or chitosan/P407 in the case of AC3) [50]. At 8 °C for Cs-CA and 6 °C for AC3, an endothermic peak is attributed to the melting of free water, whereas at −9 °C, another endothermic peak is attributed to the melting of interphasal/bound water; the latter is only present in AC3 [50]. Considering the shift in the position of the signals to lower temperatures, as well as the increase in the intensity of the signals in AC3, it can be assumed that Cur-loaded P407 micelles increase the availability of hydrogen bonds that cause the observed phase transitions, and the state of water is also modified, as the interphasal/bound water endothermic peak is only present in AC3. Therefore, MDSC measurements reflects the enthalpy changes, corresponding to the progressive formation or breakage of interactions within the gel structure, and to the structural modification of the hydrogel, as well as the melting of water at different temperatures, depending on the presence of free, interphasal, or bound freezable water by the addition of Cur-loaded P407 micelles.

### 3.5. Rheological Properties

Table 4 shows the critical deformation parameters of the Cs-CA and AC3 hydrogels. At the preparation temperature (22 °C), the systems have higher critical deformation values than those measured at the potential application temperature (37 °C). However, there are no significant differences in the neat (Cs-CA) with respect to the systems with Cur-loaded P407 micelles (AC3), neither at 22 °C nor at 37 °C. This could be due to the partial destruction of the polymer network, which reduces the capacity of the hydrogel to withstand deformation. At 37 °C, intermolecular interactions weaken, molecular mobility increases, and partial water loss may occur, all of which reduce the cohesion and strength of the hydrogel [61]. When comparing Cs-CA and AC3 systems, it is observed that the system with Cur-loaded P407 micelles (AC3) has a slightly higher critical deformation at both measurement temperatures. The presence of P407 micelles in the hydrogel can increase its critical deformation due to the stabilizing interactions that the poloxamer micelles can form with the crossed-linked Cs polymeric matrix, probably an increased number of hydrogen bonds, as evidenced by DSC measurements which indicate that interphasal/bound water increases with the presence of P407 micelles.

**Figure 4:**
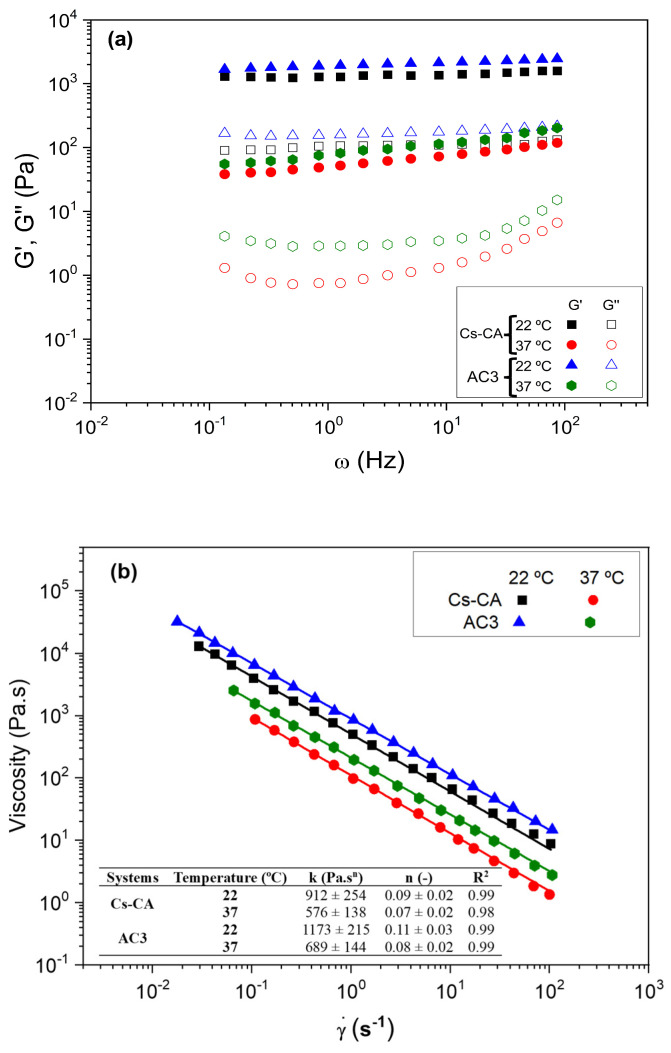
(**a**) Frequency sweep tests and (**b**) viscous flow curves performed on the different hydrogels (Cs-CA and AC3) at different temperatures (22 and 37 °C).

Figure 4a shows the profile of the elastic (G′) and viscous (G″) moduli in the studied frequency range for Cs-CA and AC3 hydrogels at the different measurement temperatures. It is observed that both systems showed a gel-like rheological behavior, where the elastic modulus (G′) was higher than the viscous modulus (G″), without large frequency variations. This is confirmed by the values of tan (δ), which are less than 1 in all cases, highlighting the contribution of the elastic moduli of the prepared systems (see Table 4). The results of the mechanical spectra indicate that both systems had sufficient stability to predict their behavior independently of the time of application of the deformation [62]. In addition, it is observed that the systems measured at 22 °C have considerably higher moduli than those measured at 37 °C (Table 4). This may be due to the fact that the polymeric network of the hydrogel is stiffer at 22 °C due to stronger interactions (such as hydrogen bonds), resulting in higher values of G′ and G″ [63,64]. At 37 °C, increased thermal energy weakens these interactions, making the network more flexible and decreasing G′ and G″. The lower mobility of water molecules at 22 °C also contributes to stiffness. In addition, possible phase transitions at 37 °C can reduce the cross-link density and increase the fluidity of the hydrogel. On the other hand, the AC3 hydrogel shows slightly higher rheological moduli. This can be explained by the influence of P407 micelles on the structure and properties of the hydrogel due to increased hydrogen bond interactions with the cross-linked Cs matrix.

Figure 4b shows the flow curves of Cs-CA and AC3 hydrogels at 22 °C and 37 °C. The systems present a pseudoplastic behavior at both temperatures. This behavior is common in this type of material, which presents a lower flow resistance at higher deformation velocities, which has also been observed in other studies [65]. In addition, viscosities are affected by the presence of Cur-loaded P407 micelles and by temperature. Thus, systems measured at 37 °C present lower viscosity values than those measured at 22 °C. On the other hand, the AC3 hydrogel presents slightly higher viscosity values than the system that did not contain Cur-loaded P407 micelles in its composition (Cs-CA). It is important to note that these results are consistent with those obtained in the frequency sweep tests, since the biopolymeric chains are less cross-linked when the temperature increases and when they do not contain Cur-loaded P407 micelles in the composition, offering lower resistance to flow (lower viscosities); this finding supports the theory of increased hydrogen bond interactions in the optimal AC3 hydrogel. All these viscosity values have been fitted using the power law model and are included in the table inset in Figure 4b. Based on the results obtained from rheological tests, including critical deformation, elastic and viscous moduli, and viscosity, these hydrogels have suitable properties for controlled release applications, making them ideal for biomedical purposes [66].

### 3.6. Curcumin Release

**Figure 5:**
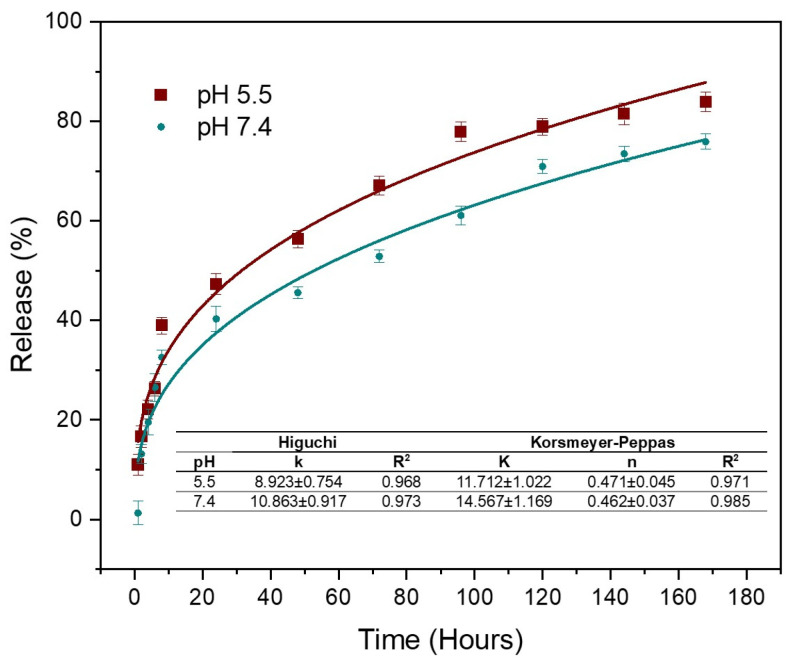
Curcumin release from AC3 hydrogel in buffer solutions at pH 7.4 and 5.5.

Figure 5 shows the results of Cur release corresponding to the AC3 hydrogel in buffer solutions at physiological pH (7.4) and slightly acidic pH (pH 5.5). The results indicate that there is a burst release of Cur occurring during the first 8 h, with 32% and 40% of Cur released at pH 7.4 and 5.5, respectively. This is followed by a sustained release, reaching 75% at physiological pH and 84% at pH 5.5 after 168 h. It is observed that at pH 5.5, the release is faster; this is because pH-sensitive compounds undergo a volume transition at different pH values, either swelling or deflating. In the case of chitosan, in acidic medium it can be protonated due to the presence of NH_2_ groups; these NH_3_^+^ units will repel each other, causing swelling in the polymer [13]. When the hydrogel contacts the medium, drug release can occur from active ingredient residues on the surface or by diffusion through the channels of the hydrogel [67]. Prolonged release is achieved for up to one week due to the second diffusion barrier for the encapsulated molecules, such as the cross-linked hydrogel [3]. The porous structure of the hydrogel acts as an efficient diffusion barrier, allowing for more sustained release [13], since the drug molecules are embedded inside the micelles, which in turn are trapped within the interconnected networks of the hydrogel [46]. The initial burst is likely due to Cur adsorbed on or near the hydrogel surface, as mentioned by Alibolandi et al. [28], who evaluated Cur-loaded micelles in dextran hydrogels. Subsequently, the process becomes more controlled. At pH 7.4, the release reached 40% within 24 h. The results show that the release of Cur from the CA-cross-linked Cs hydrogel follows the Korsmeyer–Peppas model, with an exponent n very close to 0.5, indicating anomalous transport; that is, in addition to a diffusion mechanism, a more complex mechanism also participates in the release [34]. This suggests that Cur is released proportionally to the square root of time, implying a rapid initial release that progressively slows down. This behavior is consistent with a stationary hydrogel matrix, where drug release is primarily due to diffusion from the interior of the gel into the surrounding medium. Diffusion-controlled release is beneficial for therapeutic applications, as it allows for a sustained and predictable release of Cur, improving its bioavailability and therapeutic efficacy while minimizing the need for frequent administration. Furthermore, these results have been compared with the Huggins model, confirming that the Korsmeyer–Peppas model provides a better fit to describe the release kinetics in our Cs hydrogel system.

These results agree with the work by Shah et al. [17], who developed a hydrogel based on Cs with carboxymethyl cellulose and poloxamer F 127; their systems presented a burst release of 50% in 8 h, followed by a sustained release during 24 h. It was concluded that above 24 h, the system can be considered promising for biomedical applications. Similarly, Das et al. [46] obtained a composite based on alginate/Cs and poloxamer encapsulated Cur, observing an improvement in the encapsulation percentages when poloxamer micelles were added to the system due to the encapsulation of the drug inside the micelles. Liu et al. [8] obtained hydrogels based on Cs loaded with Cur incorporated into oligoconjugated linoleic acid vesicles, obtaining a burst of around 10% and release percentages between 28 and 51% within 96 h.

### 3.7. In Vitro Biocompatibility Evaluation

For the in vitro biocompatibility tests, analyses were performed using the blank Cs-CA hydrogel, and the sample designated AC3, which consists of the hydrogel with Cur-loaded P407 micelles.

Hemolysis, the rupture of red blood cells with subsequent hemoglobin release, is used to evaluate biocompatibility. It can occur upon contact with an external agent under testing, causing the rupture of blood cells [68]. Erythrocytes typically exhibit a biconcave, disk-like shape under normal conditions; however, alterations to their cell membrane can result in a change in shape and subsequent hemolysis [66].

**Figure 6:**
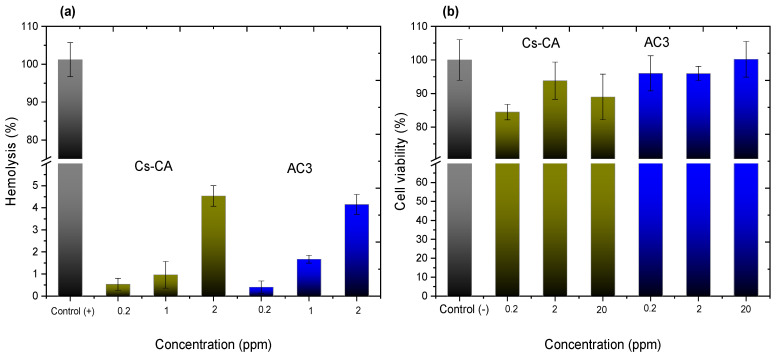
(**a**) Hemolysis percentages for Cs-CA and AC3 samples at 0.2, 1, and 2 ppm. All data are presented as the mean value ± SD, where *n* = 5; (**b**) cell viability percentages for Cs-CA and AC3 samples at 0.2, 2, and 20 ppm after 24 h of exposure in VERO CCL-81 cell cultures. All data are presented as the mean value ± SD, where *n* = 3.

Figure 6a shows the graphs representing the percentage of hemolysis caused by the blank hydrogel cross-linked with citric acid (Cs-CA) and the cross-linked hydrogel containing Cur-loaded P407 micelles (AC3). In accordance with ASTM F756 [69], Standard Practice for Assessment of Hemolytic Properties of Materials, materials with hemolysis percentages above 5% are considered hemolytic, 2–5%, slightly hemolytic, and below 2%, non-hemolytic. In the case of sample Cs-CA, hemolysis percentages of 0.54%, 0.95%, and 4.53% are presented at concentrations of 0.2, 1, and 2 ppm, respectively, when poloxamer micelles loaded with curcumin are added to the hydrogel, which presents hemolysis percentages of 0.41%, 1.67%, and 4.15%, considered non-hemolytic (0.2 and 1 ppm) and slightly hemolytic at a concentration of 2 ppm, indicating that the hydrogels can remain in contact with the erythrocyte without causing a severe hemolytic reaction at such concentrations. Single-factor ANOVA statistical results indicated a significant difference in the percentages of hemolysis according to the concentration between treatments, obtaining a *p*-value < 0.05. Moreover, dose–response R² values of 0.950 and 0.935 were obtained for AC3 and Cs-CA, respectively. These results suggest that the concentration of the evaluated treatments has an impact on hemolysis, and the high R² values indicate a good correlation between the applied dose and the observed response for both treatments (AC3 and Cs-CA).

Ravikumar et al. [70] performed a test on the behavior of blood stored in contact with different concentrations of Cur, and mentioned that the addition of this active ingredient is beneficial for erythrocytes, and that it maintains the stability of hemoglobin, since it protects it from oxidation. Cur also prevents the generation of reactive oxygen species and reduces osmotic fragility and hemolysis. Jafari et al. [71] obtained a pH stimulus-sensitive system of laponite RD-Cs and polyvinyl alcohol, evaluated in concentrations between 25 and 800 µg/mL, presenting a maximum hemolysis percentage of 7.5%, and it was concluded that their systems can be considered as hemocompatible. Likewise, some surfactants, such as poloxamers, can have a protective effect on erythrocytes because, as a consequence of their interaction, the balance of ion concentration inside and outside the cell is modified, thus reducing hemolysis [72]. In some of the potential applications of hydrogels, such as wound dressings, anti-hemolytic properties are highly desirable, since hemolysis can negatively affect the wound healing process [73].

Another methodology to evaluate biocompatibility is the MTT cytotoxicity assay, a colorimetric study. In this assay, the MTT salt 3-(4,5-dimethyl-2-thiazolyl)-2,5-diphenyl-2H tetrazolium bromide is reduced to formazan by the enzyme succinate dehydrogenase. This in turn produces a color change from yellow to purple, which can be quantified [74].

Evaluating the behavior of materials in cell lines is important for understanding their interaction and cytotoxicity. VERO CCL-81 cells are a stable and standard model for cytotoxicity testing [37].

Figure 6b presents viability graphs with the VERO CCL-81 cell line of the Cs-CA and AC3 hydrogels at 0.2, 2, and 20 ppm; viability percentages of 84%, 93%, and 88% are obtained for the blank Cs hydrogel cross-linked with CA. The percentages of cell viability increase to 96%, 95%, and 100% when P407 micelles loaded with Cur are added to the hydrogel (AC3 sample). Single-factor ANOVA statistical results do not show significant differences in cell viability between the groups treated with different concentrations of hydrogels (0.2, 2, and 20 ppm) and the control (*p* > 0.05 for all pairwise comparisons). This indicates that, under the experimental conditions tested, none of the tested concentrations of hydrogels resulted in a statistically significant change in cell viability compared to the control. In the hydrogel with Cur-loaded P407 micelles, greater cell viability was obtained; P407 could not only be considered non-toxic, but it also has a protective effect because it is a poloxamer with a high hydrophilic proportion [75]. Furthermore, according to the report by Facchi et al. [76], who obtained N,N-dimethyl chitosan and N,N,N-trimethyl chitosan nanoparticles loaded with Cur and evaluated their cytotoxic effect with VERO CCL-81 cells, the systems loaded with Cur presented a higher cell viability.

## 4. Conclusions

The hydrophobic compound Cur was successfully encapsulated in P407 micelles and incorporated into Cs hydrogels cross-linked with CA, avoiding alkaline pH conditions that could degrade it. This strategy preserved its chemical stability while enabling the formation of a robust hydrogel upon the rehydration of the cross-linked, freeze-dried formulation. The double-encapsulation approach achieved a Cur loading capacity of up to 0.38 wt%.

FTIR analysis suggested both amide bond formation and electrostatic interactions between the protonated amine (NH_3_⁺) of Cs and the carboxylate group (COO^−^) of CA, contributing to cross-linking. DSC results showed shifts in endothermic events and increased signal intensity, indicating molecular interactions between the cross-linked hydrogel and Cur-loaded P407 micelles. SEM images revealed that the incorporation of Cur-micelles significantly altered the hydrogel’s structure and texture, potentially improving drug absorption and modulating release kinetics.

Rheological analysis confirmed the gel-like behavior of all hydrogels, with the elastic modulus (G′) consistently exceeding the viscous modulus (G″) across the studied frequency and temperature range. The presence of Cur-loaded micelles in the AC3 hydrogel led to increased G′ and G″ values, and all formulations exhibited pseudoplastic flow behavior. Drug release studies showed sustained Cur release, reaching 75% at physiological pH and 84% at acidic pH after 168 h.

Biocompatibility assays demonstrated that the hydrogels were either non-hemolytic or slightly hemolytic at tested concentrations (0.2, 1, and 2 ppm) and supported high cell viability (>95%) in VERO CCL-81 cells up to 20 ppm AC3. These findings highlight the potential of these hydrogels as smart topical delivery systems for sustained drug release and wound healing applications.

## Figures and Tables

**Table 1:** Experimental conditions for obtaining cross-linked Cs hydrogels charged with Cur-loaded P 407 micelles.

Sample	CA (wt%)	Reaction Time (h)	Cs (wt%)
AC1	0.05	6	0.5
AC2	0.05	6	1
AC3	0.05	24	1
AC4	0.05	24	0.5
AC5	0.1	6	0.5
AC6	0.1	6	1
AC7	0.1	24	1
AC8	0.1	24	0.5
AC9	0.075	15	0.75
AC10	0.075	15	0.75
AC11	0.05	15	0.75
AC12	0.1	15	0.75
AC13	0.075	6	0.75
AC14	0.075	24	0.75
AC15	0.075	15	0.5
AC16	0.075	15	1
Cs-CA	0.05	24	1

AC corresponds to samples, CA to citric acid, Cs to chitosan, and Cur to curcumin.

**Table 2:** Hydrogel composition, %Cur loading capacity (*%Cur LC*), and %Cur encapsulation efficiency (*%Cur EE*) in Cs hydrogels cross-linked with CA and charged with Cur-loaded P407 micelles (hydrated gels).

Sample	% Cs (wt%)	% CA (wt%)	% P407 (wt%)	% Water (wt%)	*%Cur LC*	*%Cur EE*
AC1	14.66	0.73	34.21	50.38	0.38	67.55 ± 0.38
AC2	22.42	1.12	26.16	50.29	0.29	75.69 ± 0.43
AC3	22.42	1.12	26.16	50.29	0.29	80.07 ± 0.45
AC4	14.66	0.73	34.21	50.38	0.38	69.01 ± 0.39
AC5	14.45	1.44	33.72	50.38	0.38	66.86 ± 0.38
AC6	21.92	2.19	25.58	50.28	0.28	70.38 ± 0.40
AC7	21.92	2.19	25.58	50.28	0.28	74.91 ± 0.42
AC8	14.45	1.44	33.72	50.38	0.38	70.44 ± 0.40
AC9	18.87	1.41	29.37	50.33	0.33	69.93 ± 0.40
AC10	18.87	1.41	29.37	50.33	0.33	66.76 ± 0.38
AC11	19.05	0.95	29.65	50.33	0.33	70.01 ± 0.40
AC12	18.70	1.87	29.09	50.32	0.32	71.71 ± 0.41
AC13	18.87	1.41	29.37	50.33	0.33	72.48 ± 0.41
AC14	18.87	1.41	29.37	50.33	0.33	71.39 ± 0.41
AC15	14.55	1.09	33.96	50.38	0.38	68.03 ± 0.39
AC16	22.17	1.66	25.87	50.29	0.29	78.33 ± 0.44
Cs-CA	47.61	2.38	0	50.00	-	-
Cs-CA-Cur Ace	47.61	2.38	0	50.00	-	-
P407-Cur	0.00	0.00	26.16	73.84	-	-
AC3 Alk	22.93	0.00	26.76	50.30	-	-

**Table 3:** %Cur loading capacity (*%Cur LC*) and %Cur encapsulation efficiency *(%Cur EE*) reported from the literature versus this work.

System	*%Cur LC*	*%Cur EE*	Reference
Cur loaded into PEG-PLA micelles incorporated into dextran hydrogel	0.72	81.56	[28]
Cur encapsulated into liposomes coated with thiolated chitosan	0.21	88.75	[44]
Cur nano-encapsulated into fish collagen–succinyl chitosan composite hydrogel	0.23	96.2	[40]
Hybrid hydrogel made of chitosan and P123, containing gelatin and Cur	0.027	-	[45]
Composite of alginate, chitosan and P407 micelles loaded with Cur	0.10	13.0	[46]
Chitosan hydrogel cross-linked with citric acid charged with Cur-loaded P407 micelles	0.38	69.0	This work

Results consider %LC and %EE, obtained directly from the references, and in some cases calculated from experimental details given in the references.

**Table 4:** Mechanical parameters obtained for Cs-CA and AC3 hydrogels at the preparation temperature (22 °C) and at the application temperature (37 °C).

Systems	Temperature (°C)	Critical Strain (-)	G′_1_ (Pa)	Tan(δ)_1_ (-)
Cs-CA	22	0.411 ± 0.072	1211 ± 117	0.083 ± 0.011
	37	0.179 ± 0.019	67 ± 11	0.015 ± 0.003
AC3	22	0.479 ± 0.083	1898 ± 317	0.081 ± 0.013
	37	0.191 ± 0.023	83 ± 19	0.021 ± 0.005

**Figure A2:**
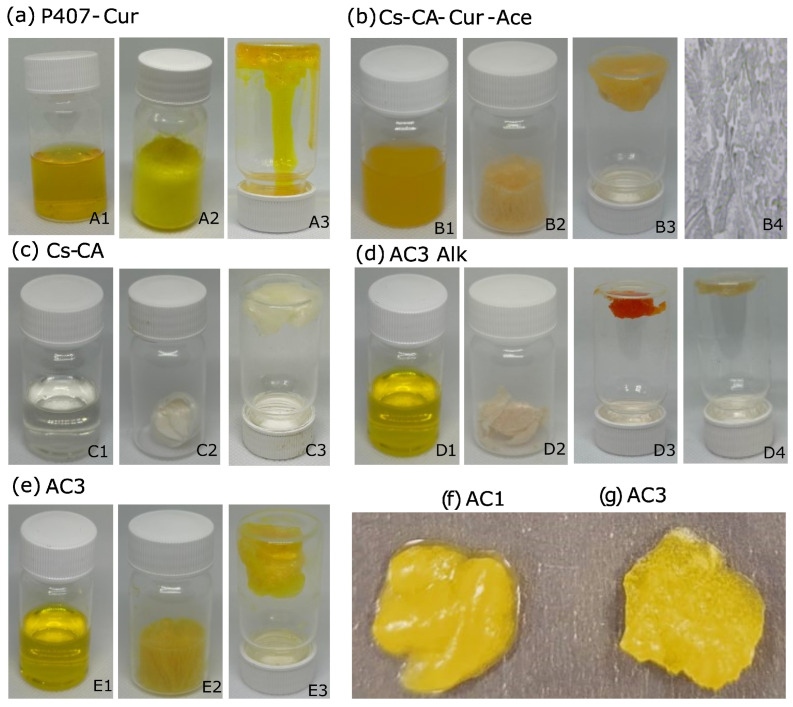
Photographs of different hydrogels and related samples. (**a**) P407-Cur, (**b**) Cs-CA-Cur-Ace, (**c**) Cs-CA, (**d**) AC3-Alk, and (**e**) AC3 hydrogel obtained under optimal conditions. All samples are shown as a solution, freeze-dried, and as a rehydrated gel. B4 optical micrograph showing crystalized Cur. D4 rehydrated gel (from freeze-dried sample shown in D2), (**f**,**g**) photographs comparing hydrogels AC1 and AC3.

## Data Availability

The original contributions presented in this study are included in this article. Further inquiries can be directed to the corresponding authors.

## Appendix A

### Appendix A.1. Calibration Curve of Curcumin

Figure A1 presents the Cur calibration curve that was used for the *%Cur EE* and Cur release calculations, showing the absorption value at 435 nm as a function of Cur concentration.

### Appendix A.2. Macroscopic Characteristics of Hydrogels and Control Samples

Figure A2 presents the physical appearance of various hydrogel samples. Some samples were prepared as controls for comparison purposes. Figure A2a (A1) shows Cur-loaded P407 (1.18%) micelles (P407-Cur); this sample was also freeze-dried (A2) and rehydrated (A3) by using enough water to keep P407 at the same concentration as in the optimal AC3 gel (~26.16% P407, ~73.84% water). Figure A2b shows a sample of cross-linked Cs loaded with Cur, but without P407 micelles (Cs-CA-Cur-Ace); for this purpose, Cur was added as a solution in acetone, followed by evaporation of the latter (B1), and then a hydrogel was obtained by freeze-drying (B2) and rehydrating (B3, ~50% water). Another sample for comparison purposes is Cs-CA, shown in A2 (Figure A2c); this was prepared by cross-linking Cs with CA (C1), subsequently freeze-dried (C2) and rehydrated (C3, ~50% water) to obtain the hydrogel. The AC3-Alk hydrogel shown in A2 (Figure A2d) was prepared by alkaline pH adjustment; for this purpose, 2.5 mL of NaOH 0.1 M was added to the solution of Cs/Cur-P407 micelles in order to cause the precipitation of Cs and the partial formation of a hydrogel with a dark orange color (D3), along with excess orange solution that did not form hydrogel; the gel part was subsequently freeze-dried (D2) and rehydrated (D4). Images in 1A (Figure A2e) show the samples obtained by the methodology taken as optimal named AC3, formed by Cs cross-linked with CA charged with Cur-loaded P407 micelles (E1), which was freeze-dried (E2) and rehydrated (E3).

The images A1, D1, and E1 show transparent yellow solutions, demonstrating effective encapsulation of Cur within P407 micelles. Conversely, in sample B1, curcumin crystallizes in the hydrogel following solvent evaporation, as revealed by the turbid appearance of this sample. The samples shown in images A2, C2, D2, and E2 were freeze-dried from the hydrogel solution. The volume of the freeze-dried Cur-loaded P407 micelles (A2) and the optimal AC3 hydrogel (E2) sample appear similar. Likewise, the volume of the freeze-dried blank sample Cs-CA (C2) and the freeze-dried Cs-CA-Cur-ACE hydrogel solution sample (B2), both based on Cs cross-linked with CA (without Cur-loaded P407 micelles), are also very similar, showing that in the absence of P407 micelles, the hydrogel solutions experiment a more pronounced shrinking upon freeze-drying. In image D2, corresponding to the freeze-dried gel formed by alkaline pH adjustment (AC3-Alk, D3) instead of cross-linking, it is observed that the hydrogel structure collapses, resulting in a compact dry solid, demonstrating the superior structural and mechanical properties of the cross-linked gel. Subsequently, the gel can be formed upon rehydration (D4), but the characteristic yellow color of Cur is lost, due to degradation induced by alkaline pH adjustment.

The images B3, C3, and E3 show rehydrated hydrogels. The optimal AC3 hydrogel (E3) maintains an unaltered yellow color, remaining fluffy with a rather expanded volume compared to controls Cs-CA and Cs-CA-Cur-Ace (C3 and B3, respectively). Additionally, Figure A2b (B4) presents the optical micrograph of the Cs-CA-Cur-Ace control hydrogel (from hydrogel B3), where precipitated Cur can be observed. Therefore, the bioavailability of Cur in this hydrogel, in which Cur was added as a solution in acetone rather than in micelles, would be compromised because it is present as a macroscopic, insoluble, dispersed form. On the other hand, when the Cur-loaded P407 micelles are rehydrated with 73.84% water (Figure A2a (A3), to achieve the same P407 concentration as in AC3), a viscous solution is obtained, confirming that hydrogel formation does not occur at this concentration of P407 in the absence of cross-linked Cs (at 25 °C). It is remarkable that all rehydrated samples shown in images A3, B3, C3, and E3 contain 1 g of sample, whilst the occupied volume is rather different, being greater in AC3, and the cross-linked Cs hydrogel containing the Cur-loaded micelles. All these observations reveal superior physicochemical, structural, and mechanical properties, as well as enhanced Cur chemical stability and homogeneity for the hydrogel formed by Cs cross-linked with CA and loaded with Cur-P407 micelles (AC3), compared to the hydrogel in which Cur was added as an acetone solution (Cs-CA-Cur-Ace), and the hydrogel formed by alkaline pH adjustment, rather than cross-linking with CA (AC3-Alk). In addition, the effect of Cs concentration is highlighted by inspecting the images in Figure A2f,g, where hydrogels AC1 and AC3, synthesized with 0.5 and 1 wt% Cs in the reaction mixture, are presented (freeze-dried and rehydrated, ~50% water). It can be observed that the hydrogel with a lower amount of Cs expels liquid upon rehydration, indicating that it is necessary to use 1 wt% Cs in order to obtain hydrogels with better structural properties, leading to increased swelling.
